# Effect of chemotherapy on cancer stem cells and tumor-associated macrophages in a prospective study of preoperative chemotherapy in soft tissue sarcoma

**DOI:** 10.1186/s12967-019-1883-6

**Published:** 2019-04-18

**Authors:** Keith M. Skubitz, Jon D. Wilson, Edward Y. Cheng, Bruce R. Lindgren, Kristin L. M. Boylan, Amy P. N. Skubitz

**Affiliations:** 1 Department of Medicine, University of Minnesota Medical School, Box 286 University Hospital, Minneapolis, MN 55455 USA; 2 Masonic Cancer Center, University of Minnesota Medical School, Minneapolis, MN USA; 3 Department of Laboratory Medicine and Pathology, University of Minnesota Medical School, Minneapolis, MN USA; 4 Present Address: Arkana Laboratories, Little Rock, AR USA; 5 Department of Orthopaedic Surgery, University of Minnesota Medical School, Minneapolis, MN USA; 6 Division of Biostatistics, University of Minnesota School of Public Health, Minneapolis, MN USA; 7 Department of Obstetrics, Gynecology, and Women’s Health, University of Minnesota School of Public Health, Minneapolis, MN USA

**Keywords:** Cancer stem cell, Sarcoma, Macrophage, Chemotherapy, PET, Positron emission tomography

## Abstract

**Background:**

Cancer stem cells (CSC) may respond to chemotherapy differently from other tumor cells.

**Methods:**

This study examined the expression of the putative cancer stem cell markers ALDH1, CD44, and CD133; the angiogenesis marker CD31; and the macrophage marker CD68 in soft tissue sarcomas (STS) before and after 4 cycles of chemotherapy with doxorubicin and ifosfamide in 31 patients with high-grade soft tissue sarcoma in a prospective clinical trial.

**Results:**

None of the markers clearly identified CSCs in STS samples. Macrophages represented a prominent component in viable tumor areas in pre-treatment STS biopsies, ranging from < 5 to > 50%. Furthermore, macrophages expressed CD44 and ALDH1. Macrophage density correlated with baseline maximum standardized uptake value (SUVmax) on fluoro-deoxyglucose positron emission tomography (PET) imaging. Pre-chemotherapy CD68 staining correlated positively with the baseline SUVmax, and negatively with the percent of viable tumor cells in post-chemotherapy resection samples. In particular, cases with more CD68-positive cells at biopsy had fewer viable tumor cells at resection, suggesting a better response to chemotherapy.

**Conclusions:**

In conclusion, ALDH1, CD44, and CD133 are not likely to be useful markers of CSCs in STS. However, our observation of infiltrating macrophages in STS specimens indicates that these immune cells may contribute significantly to STS biology and response to chemotherapy, and could provide a potential target of therapy. Future studies should investigate macrophage contribution to STS pathophysiology by cytokine signaling.

**Electronic supplementary material:**

The online version of this article (10.1186/s12967-019-1883-6) contains supplementary material, which is available to authorized users.

## Background

The response of soft tissue sarcoma (STS) to chemotherapy and long-term outcome are difficult to predict, and most patients with metastatic disease die from disease. Most tumors are composed of a population of tumor cells capable of continued cell division, commonly termed cancer stem cells (CSC) [1, 2], and a population of tumor cells that have undergone changes such that they no longer have the capacity to divide and form new cells capable of further cell division. Various normal host cells are also present.

The idea of directing treatment against CSCs has long been discussed [1, 3, 4], and was supported by the classic studies of Skipper et al. in the 1960s in which tumorigenic cells could also be termed CSCs [5]; to cure cancer, it is thought that all CSCs must be eliminated. Clinical trials suggest a role for CSCs in some solid tumors. ALDH1, CD44, and CD133 are among the putative CSC markers described [6–11]. In some cases the proportion of ALDH1+ cells has been reported to increase in breast cancer patients receiving neoadjuvant chemotherapy [6, 9]; in one study, those patients whose post-chemotherapy tumors showed an increase in ALDH1 + tumor cells had shorter disease-free survival than other patients [6]. However, the utility of ALDH1 as a marker of CSCs is not clear in some solid tumors, such as ovarian cancer; in fact, gene expression studies have reported that ALDH1A1 expression was up-regulated in normal ovary, benign ovarian tumors, and borderline ovarian carcinomas as compared with high-grade malignant ovarian carcinoma [12–14]. CD133 has been suggested as a CSC marker in a number of tumors (reviewed in [11]), and high expression has been correlated with poor overall survival in embryonal rhabdomyosarcoma [11]. However, the role of CSCs in STS biology and treatment response is poorly understood.

Like G_0_ cells that are not replicating, CSCs may be more resistant to chemotherapy than other cells in the tumor. We hypothesized that in patients with STS treated with chemotherapy and surgery, the number of CSCs in the residual tumor would predict treatment outcomes. We recently completed a prospective clinical trial designed to examine the utility of fluoro-deoxyglucose positron emission tomography combined with computerized axial tomography (FDG PET-CT) imaging to predict response to chemotherapy in STS [15]. This trial examined PET imaging characteristics and histopathology before and after pre-operative chemotherapy with doxorubicin and ifosfamide in 69 patients with STS.

In the present study, we used immunohistochemistry (IHC) to detect biomarkers of CSCs in tumor samples before and after pre-operative chemotherapy. We examined the expression of three markers (ALDH1, CD44, and CD133) previously described as markers of CSCs [6–11, 16]. In addition, we quantified the expression of CD31, an endothelial marker of angiogenesis [17, 18], to determine whether angiogenesis predicts treatment resistance. We observed that CD44 and ALDH1 were also expressed by macrophages, and we therefore conducted IHC studies using CD68 to specifically identify the proportion of tumor macrophages. Macrophages were found to represent an unexpectedly prominent component in many of the STS cases. Macrophages have many functions and can secrete a variety of cytokines capable of paracrine signaling and could play an important role in STS biology.

## Methods

### Study design

The study was approved by the University of Minnesota IRB. Thirty-one patient samples were collected before chemotherapy by biopsy and after chemotherapy at the time of primary excision, during our prospective clinical trial of PET imaging in STS [15] (Additional file 1: Table S1). Samples were obtained from 2006 to 2014 from patients ≥ 16 years old with high-grade (FNCLCC grade 3) STS of the extremities or body wall whose tumors were greater than 5 cm in maximum diameter. Patients received pre-operative chemotherapy followed by wide surgical excision of their tumor and subsequent external beam radiation; the goal of the clinical trial was to correlate treatment response with early PET changes [15]. The preferred chemotherapy regimen was pegylated-liposomal doxorubicin (PLD) at 45 mg/m^2^ intravenously (IV) on day 1 every 28 days, with ifosfamide given by continuous intravenous infusion (CIVI) at 1.5 g/m^2^/day for 6 days (total dose over 6 days of 9 g/m^2^), in conjunction with mesna 1.5 g/m^2^/day for 7 days [19]. In some early cases, free doxorubicin was used because of insurance restrictions at a dose of 9.3 mg/m^2^/day by continuous IV infusion over 7 days (total dose over 7 days of 65 mg/m^2^). For both the PLD and free doxorubicin regimens, granulocyte colony stimulating factor (G-CSF) was used prophylactically. FDG PET-CT was performed at baseline before chemotherapy and after cycle 4 immediately before wide surgical excision of the tumor as previously described [15]. External beam radiation was administered to patients after wide surgical excision, and patients were then followed for recurrence.

### Immunohistochemistry

IHC was performed using standard techniques following the manufacturer’s instructions with antibodies to ALDH1 (BD Clone 44, BD Transduction Laboratories) and CD133 (clone 7, kindly provided by Dr. John Ohlfest, University of Minnesota). IHC for CD31, CD44, and CD68 was performed by a CLIA-certified laboratory (University Hospital, Minneapolis) using the antibodies JC70 (CD31) (Santa Cruz Biotechnology), DF1485 (CD44, Santa Cruz Biotechnology), and KP-1 (CD68) (Santa Cruz Biotechnology). Stained slides were reviewed by a pathologist with expertise in sarcomas (JDW). For CD31, staining was scored on a scale of 0–3 (reflecting vessel density). For CD68, staining was scored at 100× magnification as the percent of cells, including stroma, that were positive: 0–5%, > 5–25%, > 25–50%, > 50–75%, or > 75%. For CD133, staining was scored as the percent of tumor cells positive, on a scale of 0, 1–33% positive (score 1), 34–66% positive (score 2), or > 67% positive (score 3). Colon was used as a positive control for CD133 expression. For ALDH1 and CD44, staining was scored both as a percent of tumor cells positive on a scale of 0, 1–33% positive (score 1), 34–66% positive (score 2), or > 67% positive (score 3) and also for intensity of staining as 0, 1, 2, or 3.

### Histologic grading

Grading of percent necrosis was performed by assessing the percentage of viable tumor and percentage of non-viable tumor; the latter specifically addressed the extent of necrosis, fibrosis, hemorrhage, and cystic change, each in percentages. This assessment was obtained by correlating the gross features and the histologic sections. In addition, each case was assigned a specific histologic diagnosis and histologic grade according to the NCI system and the FNCLCC system. Histologic response percentage was defined as 100 − percentage of viable remaining tumor.

#### Statistical methods

Due to the ordinal ranking of the CSC markers, non-parametric Spearman rank correlation coefficients were used to measure their association with quantitative clinical outcomes, including baseline SUVmax, post-chemotherapy SUVmax, percent change in SUVmax, percent of viable tumor cells and change in tumor size. Correlations greater than 0.3 or less than − 0.3 were considered to be of interest and were reported in the results. In most cases, the corresponding p-values reached a level of statistical significance of less than 0.1, except where the sample size was very low. The non-parametric Wilcoxon rank sum test compared the two groups defined by synovial sarcoma vs. all others, and by alive vs. dead based on the follow-up time post-surgery up to 9.4 years. p-values less than 0.1 were determined to be statistically significant for these two-sample tests.

## Results

### Stem cell markers in STS

Staining of CD133, ALDH1, CD44, CD31, and CD68 was available in up to 30 pre-chemotherapy biopsy samples and up to 28 post-chemotherapy resection samples (Additional file 1: Table S1 and Additional file 2: Table S2).

**Fig. 1:**
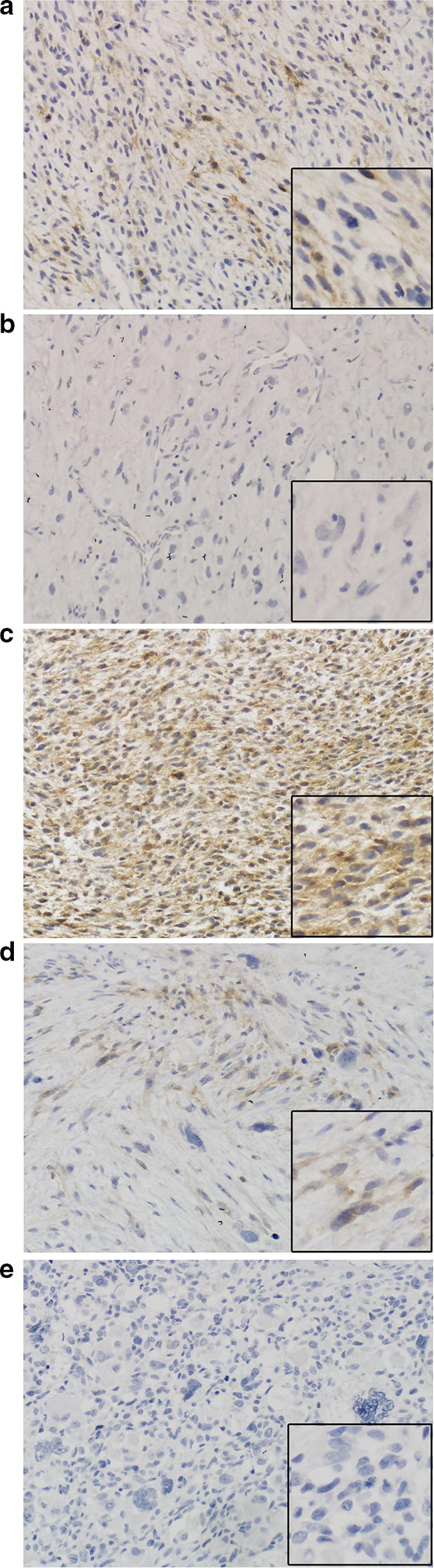
Representative staining intensity scores for CD133 at ×200. **a** Case 19, pre-treatment biopsy, score 1; **b** case 19, post-treatment resection, score 0; **c** case 10, pre-treatment biopsy, score 3; **d** case 10, post-treatment resection area with retained spindle cells, score 1; **e** case 10, post treatment resection area with more epithelioid and multinucleate tumor cells, score 0. Higher magnification inserts are shown in the bottom right of each panel

CD133 expression was detected in only 2 of the 25 pre-chemotherapy biopsy samples (cases 10 and 19, Additional file 2: Table S2), with representative images shown in Fig. 1. In case 10 at baseline, 66–100% of tumor cells were positive at a score of 1, while 33% of the tumor cells had a score of 3; after chemotherapy 33% had a score of 1, but only in the cells that retained spindle characteristics.

**Fig. 2:**
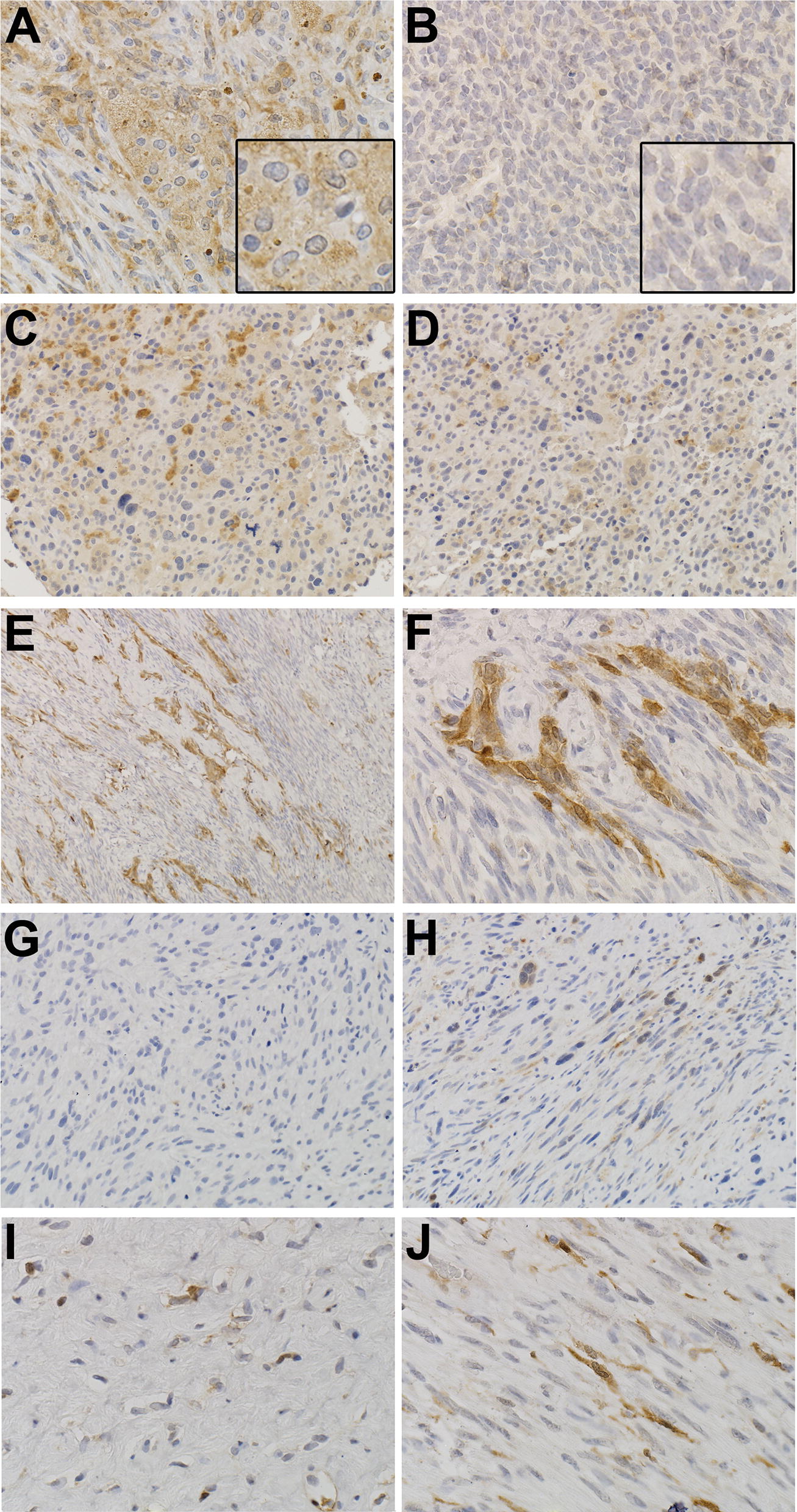
Representative staining intensity scores for ALHD1. **A** Macrophage staining at ×400. **B** (×400), case 4, pretreatment biopsy, score 0. **C**, **D** Case 5, pretreatment biopsy, scores 0 (stained cells are macrophages). **E** (×100) and **F** (×400), case 4, post treatment, score 1 for percent of tumor cells positive/score 2 for staining intensity of tumor cells (few to no macrophages are present). **G** (×200), case 16 pretreatment biopsy, score 0; **H** (×200), case 16 post treatment resection, score 1 for percent of tumor cells positive/2 for staining intensity. **I** (×400), case 20 pretreatment biopsy, score 1 for percent of tumor cells positive/1 for staining intensity; **J** (×400), case 20 post-treatment resection, score 1 for percent of tumor cells positive/3 for staining intensity. Higher magnification inserts are shown in the bottom right of **A** and **B**

ALDH1 staining was observed in 11 of the 30 pre-treatment samples and was generally similar in pre- and post-chemotherapy specimens (Additional file 2: Table S2). In 1 synovial sarcoma, only cells with epithelioid morphology were positive, while the spindle cell component was negative. ALDH1 expression in tumor cells ranged from 0 (Fig. 2B–D) to prominent staining (score 3). Of the 14 evaluable paired cases after chemotherapy, ALDH1 expression scores in tumor cells increased in 4, were unchanged in 7, and decreased in 3 (Additional file 2: Table S2). In addition to staining some tumor cells, ALDH1 was also found to be prominently expressed in macrophages, as has been reported [20–22] (Fig. 2A).

**Fig. 3:**
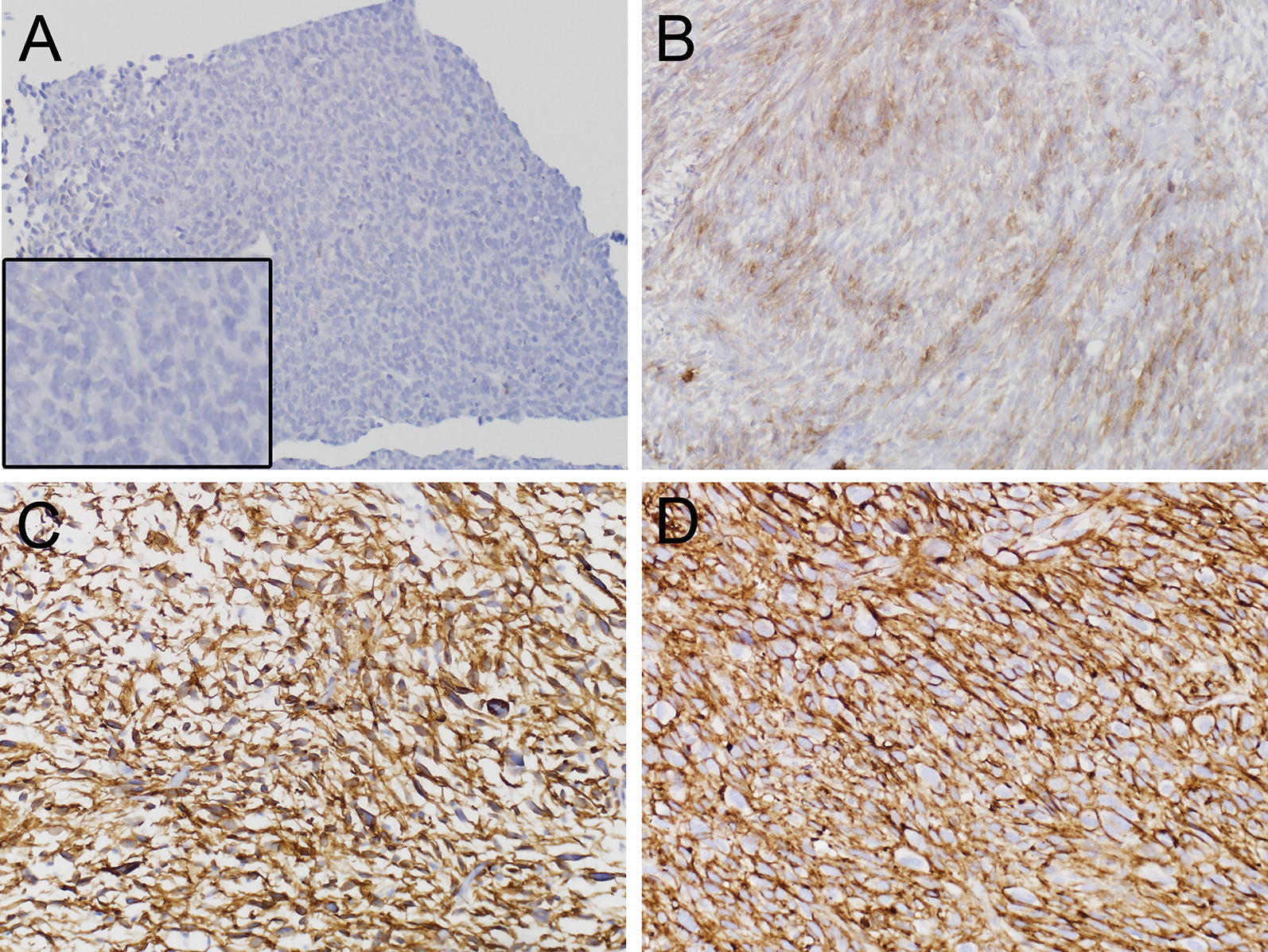
Representative staining intensity scores for CD44. **A** (×200), case 4, pretreatment biopsy, score 0; higher magnification insert is shown in bottom left. **B** (×200), case 4, post treatment resection, score 2 for percent tumor cells positive/score 2 for staining intensity. **C**, **D** (×200), case 13 pretreatment biopsy, score 3 for percent of tumor cells positive/3 for staining intensity of tumor cells

CD44 staining of tumor cells was seen in 26 of 30 pre-chemotherapy samples. Tumor cell expression of CD44 ranged from no expression (Fig. 3A) to strong expression (Fig. 3C, D). CD44 expression increased in tumor cells after chemotherapy in 7 cases, was unchanged in 15 cases, and decreased in 2 cases of the 24 evaluable paired cases. CD44, like ALDH1, also stained macrophages, as expected [23, 24] and can be seen in Fig. 3A.

### Macrophage infiltration in STS tumors

**Fig. 4:**
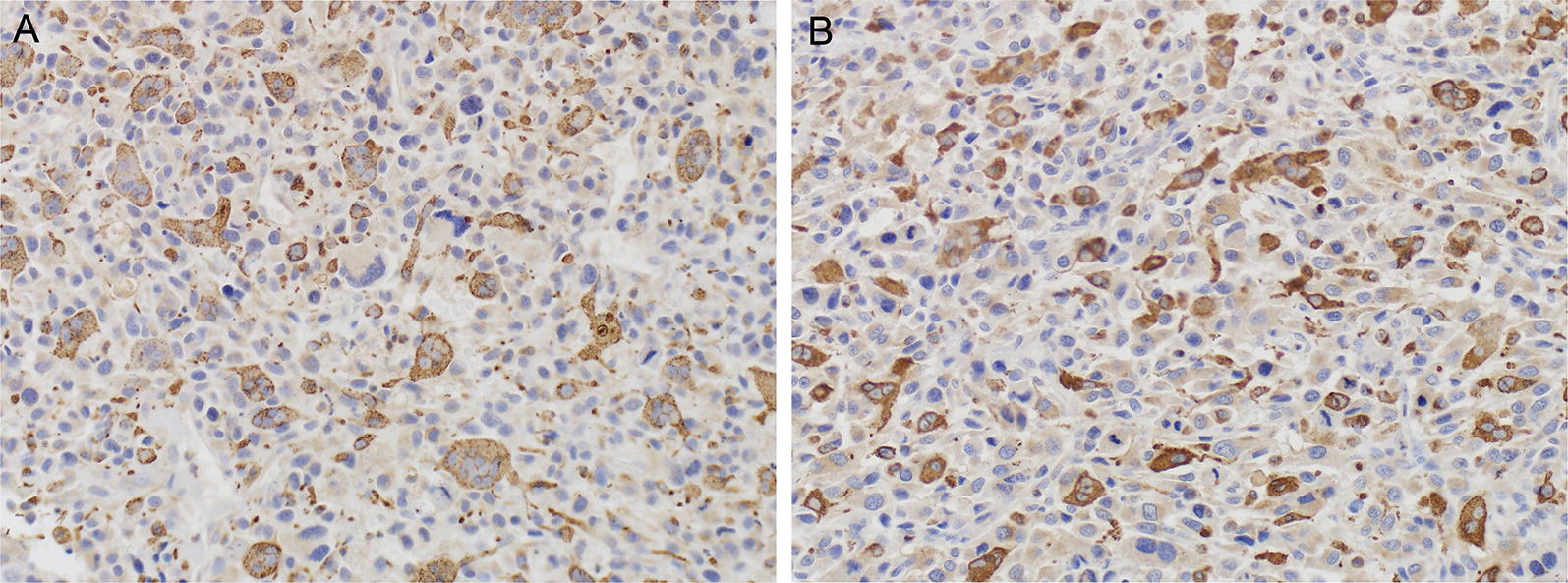
Representative staining intensity scores for CD68 at ×200. **A** Pretreatment biopsy, score > 25–50% of cells positive; **B** post treatment resection, score > 25–50% of cells positive. The stained cells are macrophages, while the tumor cells exhibit faint background staining

As described above, significant staining of macrophages for both CD44 and ALDH1 was observed, as has been reported [20, 22–24], and macrophage infiltration of the tumors was more prominent than expected. We therefore performed IHC with CD68 to better quantify the degree of macrophage infiltration of tumor specimens. Some areas had more macrophages than others, and there were more macrophages in areas of tumor necrosis. We quantified CD68 positivity in both viable and necrotic regions of pre-chemotherapy biopsy specimens and post-chemotherapy resection specimens, and confirmed that the density of macrophages was greater, and often markedly so, in necrotic areas (not shown). Staining for CD68 was scored as the percent of all cells, including stromal cells and macrophages, that were positive. A representative image showing 25–50% staining is shown in Fig. 4. In general, necrotic regions contained 75–100% CD68 + macrophages in both the pre-and post-chemotherapy specimens. In some cases, nearly 100% of the remaining cells in necrotic areas after chemotherapy were macrophages. Since macrophages could be recruited to the tumor both by factors actively secreted by tumor cells, and also by the products of necrosis, it was not surprising to find many macrophages in necrotic regions. Subsequent detailed analyses of CD68-positive cells were performed only on viable regions of tumor.

Macrophages comprised from < 5% to, in some cases, 50–75% of cells present in viable regions of the tumor in the 29 paired pre-chemotherapy biopsy specimens. Only 4 of the 29 evaluable samples had 0–5% macrophages (2 synovial sarcoma, 1 leiomyosarcoma, and 1 malignant peripheral nerve sheath tumor), 9 had 5–25% macrophages (5 UPS, 2 synovial sarcoma, 1 fibrosarcoma, 1 spindle cell sarcoma), and 11 had 25–50% macrophages (7 UPS, 1 synovial sarcoma, 1 fibrosarcoma, 2 dedifferentiated liposarcoma), while 4 tumors were composed of 50–75% macrophages (4 UPS) in the pre-chemotherapy viable tumor (Additional file 2: Table S2). Thus, macrophage infiltration was most prominent in UPS. After treatment, the number of macrophages in viable tumor areas increased in some cases and decreased in others without a clear pattern (Additional file 2: Table S2). Macrophage density in pre- and post-chemotherapy tumor specimens was variable but prominent in many samples (Additional file 2: Table S2).

### CD31 staining for angiogenesis in STS tumors

**Fig. 5:**
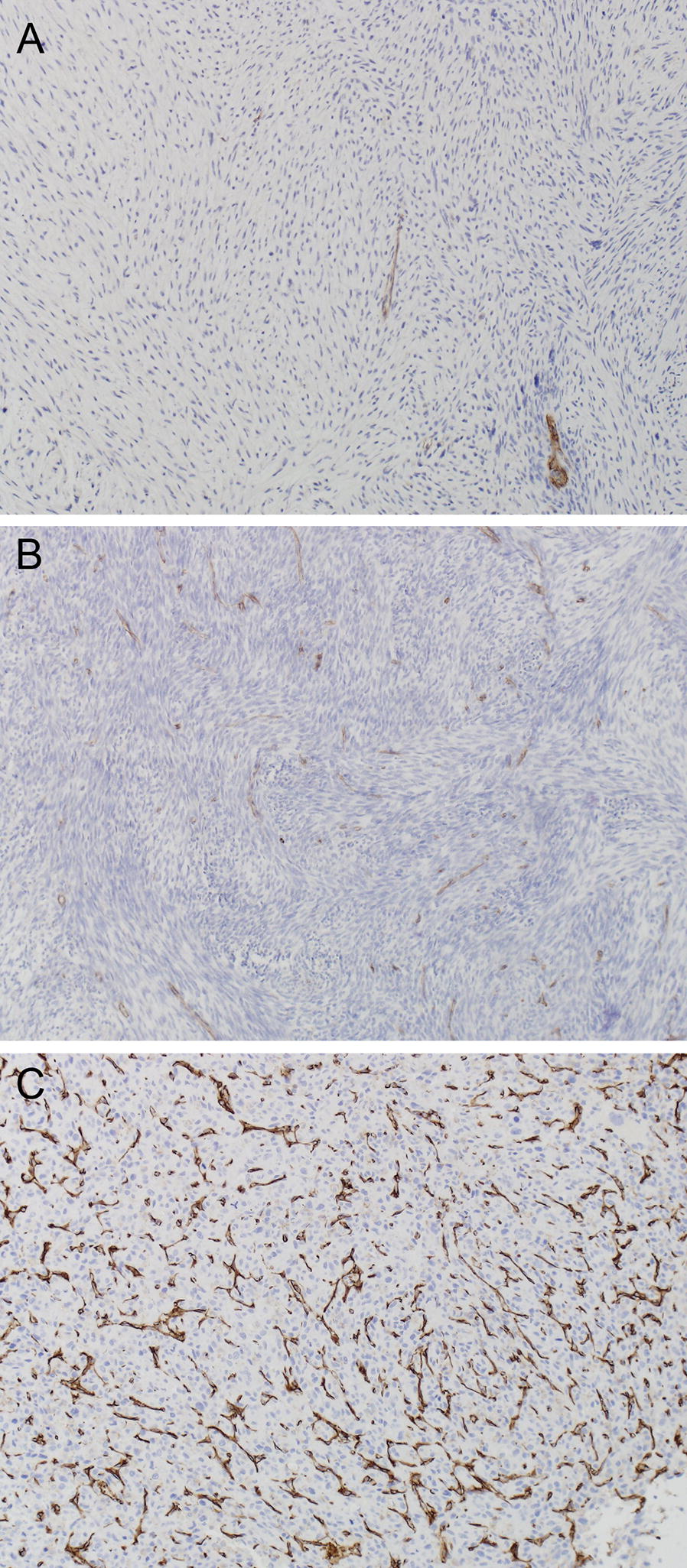
Representative staining intensity scores for CD31 at ×100. **A** Score 1; **B** score 2; **C** score 3

We also evaluated angiogenesis by IHC staining for the endothelial marker CD31. CD31 staining was detected in all 28 of the pre-treatment specimens and was generally similar in the 20 paired pre-and post-chemotherapy specimens evaluated (Additional file 2: Table S2). CD31 staining increased in 5 cases, remained the same in 14 cases, and decreased in 1 case (Additional file 2: Table S2). Representative staining for CD31, reflecting vessel density, is shown in Fig. 5.

### Correlation of IHC and outcomes

Possible associations between IHC staining and outcomes from our prospective PET imaging study [15] were examined. Specifically, baseline and post-chemotherapy ALDH1 (% positive and intensity), CD44 (% positive and intensity), CD133, CD31, and CD68 were tested for correlation with baseline (pre-chemotherapy) SUVmax, post-chemotherapy SUVmax, % change in SUVmax, diagnosis (synovial vs other), survival, percent viable cells post-chemotherapy, and change in tumor size (Table 1). Correlations between CD31 and CD68 were also tested.

**Table 1:** Correlations between IHC markers and tumor characteristics and survival

	Baseline SUVmax	Post-chemo SUVmax	% change in SUVmax	Synovial vs other^a^	% viable cells post-chemo	Change in tumor size	Survival
Baseline IHC							
ALDH1% positive, n = 30	No	No	No	No	No	No	No
ALDH intensity, n = 30	No	No	No	No	No	No	Higher in alive, p = 0.100
CD44% positive, n = 30	No	R = 0.46, p = 0.014	No	Lower in SS p = 0.005	No	No	No
CD44 intensity, n = 30	R = 0.36, p = 0.048	R = 0.36, p = 0.057	No	Lower in SS p = 0.073	No	No	No
CD68% positive, n = 28	R = 0.38, p = 0.044	No	No	Lower in SS p = 0.058	R = − 0.37, p = 0.051	No	No
CD31% positive, n = 27	No	R = − 0.39, p = 0.051	R = 0.45, p = 0.022	No	R = − 0.30, p = 0.126	R = − 0.55, p = 0.004	No
Post-chemotherapy IHC							
ALDH1% positive, n = 14	No	No	No	No	R = 0.32, p = 0.260	R = 0.41, p = 0.166	No
ALDH intensity, n = 14	No	No	No	No	R = 0.39, p = 0.168	R = 0.54, p = 0.055	No
CD44% positive, n = 24	R = 0.34, p = 0.101	R = 0.44, p = 0.043	No	Lower in SS, p = 0.010	No	R = − 0.39, p = 0.073	No
CD44 intensity, n = 24	R = 0.50, p = 0.013	No	No	No	No	R = − 0.42, p = 0.049	No
CD68% positive, n = 26	R = 0.36, p = 0.068	R = 0.34, p = 0.105	No	Lower in SS, p = 0.004	R = − 0.44, p = 0.023	No	No
CD31% positive, n = 22	No	R = − 0.30, p = 0.197	R = 0.38, p = 0.096	No	No	R = 0.46, p = 0.036	No
Change for post- minus pre-chemotherapy IHC							
ALDH1% positive, n = 14	No	No	No	No	No	R = 0.38, p = 0.199	No
ALDH1 intensity, n = 14	No	No	No	No	R = 0.31, p = 0.285	R = 0.48, p = 0.098	No
CD44% positive, n = 24	No	No	No	No	No	No	No
CD44 intensity, n = 24	No	R = − 0.40, p = 0.066	No	Higher in SS, p = 0.050	No	No	No
CD68% positive, n = 24	No	No	No	Lower in SS, p = 0.046	No	No	No
CD31% positive, n = 20	No	No	R = − 0.35, p = 0.155	No	No	No	No

A “no” value indicates no association as defined by an R value > − 0.03 and < 0.3; SS

^a^Synovial sarcoma

For CD133, there were too few non-zero values for statistical analysis. However, both cases that showed CD133 staining at baseline demonstrated excellent chemotherapy effect based on tumor cell viability.

ALDH1 did not correlate strongly with the measured outcomes, although some correlations were found (Table 1). For example, post-treatment ALDH1 staining intensity correlated positively with the change in tumor size; i.e., more intense ALDH1 staining correlated with tumor growth, and pre-chemotherapy ALDH1 staining intensity correlated weakly negatively with survival; specifically, those who died had a lower intensity of ALDH1 staining. The percent of pre-chemotherapy ALDH1-positive cells did not correlate with any endpoints in the analysis. The percent of post-treatment ALDH1-positive cells was weakly positively correlated with the percent of viable tumor cells and with change in tumor size. In other words, tumor growth tended to be higher in tumors with more ALDH1-positive cells.

The percent of pre-chemotherapy CD44-positive cells was positively correlated with post-chemotherapy SUVmax. The percent of post-treatment CD44-positive cells was positively correlated with baseline SUVmax and post-treatment SUVmax; and negatively correlated with tumor size. Synovial sarcomas had a lower percent of both pre-chemotherapy and post-treatment CD44-positive cells than the other sarcomas.

Pre-treatment CD44 staining intensity was positively correlated with baseline SUVmax and post-treatment SUVmax. Pre-chemotherapy synovial sarcomas had lower CD44 staining intensity than other sarcomas. Post-treatment CD44 staining intensity was positively correlated with baseline SUVmax and negatively correlated with change in tumor size.

Pre-chemotherapy CD68 staining correlated positively with baseline SUVmax, and negatively with the percent of viable tumor cells in post-chemotherapy resection samples. In other words, cases with more CD68+ cells at biopsy had fewer viable tumor cells at resection, suggesting a better response to chemotherapy. Synovial sarcomas had lower CD68 staining when compared with all other diagnoses.

Post-treatment CD68 staining correlated positively with baseline SUVmax and post-treatment SUVmax (pre-operative SUVmax), and correlated negatively with the percent of viable tumor cells in the resection specimen. Synovial sarcomas had lower CD68 staining than other sarcomas.

Baseline (pre-chemotherapy) CD31 staining was negatively correlated with post-chemotherapy SUVmax, and positively correlated with the percent change in SUVmax from baseline; in other words, a lower CD31 at baseline correlated with a smaller drop in SUV from pre-chemotherapy to post-chemotherapy. Baseline CD31 also correlated positively with change in tumor size (i.e., more tumor growth and higher baseline CD31). Post-treatment CD31 staining correlated positively with change in SUVmax and change in tumor size. Baseline CD31 did not correlate with baseline or post-chemotherapy CD68.

### Changes in marker staining after chemotherapy

Correlations of the changes in expression of the markers examined between pre- and post-chemotherapy specimens are shown in Table 1. The post- minus pre-chemotherapy for ALDH1-positive cells was weakly positively correlated with change in tumor size, such that an increase in ALDH1 positive cells was associated with an increase in tumor size. The post- minus pre-chemotherapy increase in ALDH1 staining intensity was positively correlated with percent of viable tumor cells in the resection specimen and change in tumor size. Change in the percent of CD44-positive cells showed no correlation with measured outcomes. Post-minus pre-treatment increase in CD44 staining intensity was negatively correlated with post-treatment SUVmax, and was higher in synovial sarcomas than the other diagnoses. Post- minus pre-chemotherapy increase in CD31 staining was negatively correlated with the percent increase in SUVmax. Post– minus pre-treatment CD68 staining scores were lower in synovial sarcomas than other sarcomas. There was no correlation between the baseline or post-treatment CD31 and CD68.

## Discussion

CSCs have been extensively studied in many cancers [1, 2, 6–14, 16, 25–38], with ALHD1, CD44, and CD133 among the most investigated CSC markers [39, 40]. Studies using flow cytometry or IHC have reported that CSC comprise from 0.1 to 20% of cells in some tumors [39]. Our study examined ALHD1, CD44, and CD133 expression before and after preoperative chemotherapy in a cohort of STS cases enrolled on a prospective clinical trial to assess whether FDG PET-CT imaging predicts response to neoadjuvant PLD and ifosfamide [15]. Our results did not support the initial hypothesis that IHC staining for ALHD1, CD44, and CD133 would detect significant changes in CSC between pre-and post-treatment STS samples. Detection of CSCs with ALDH1 and CD44 was not straightforward because tumor-infiltrating macrophages were often a prominent component of the tumor and they also expressed CD44 and ALDH1.

The concept of CSCs is further complicated in that CSCs and non-CSCs are not necessarily “hardwired”; in some cases, non-CSCs may become CSCs, and CSCs may convert to non-CSCs [1]. In addition, while CSCs do not have to be resting or G0 cells [1], if “non-CSCs” were more resistant to a treatment when in G0 phase, they might functionally appear as “true CSCs.”

In many systems, the inflammatory response may have a dramatic effect on tumor growth [41–43]. In our study, CD68-positive macrophages were found to represent a prominent component of the viable tumor regions of many of the STS cases. Macrophages express CD44 [23, 24, 44], and inflammation up-regulates ALDH1 expression in some macrophages [22]. Our finding of many macrophages in the necrotic tumor regions was not surprising, as monocyte-derived macrophages can sense multiple signals reflecting tumors and rapidly accumulate in inflamed tissue (reviewed in [45] and [46]). Tumor associated macrophages (TAMs) exist on a spectrum, from a pro-inflammatory M1 to a pro-tumor M2 state and are not static [45, 47, 48]. For example, macrophages can vary in phenotype from producing growth-inhibiting nitric oxide to growth-promoting ornithine, both derived from arginine via inducible nitric oxide synthase or arginase, respectively [49]. CSF1 recruits macrophages, and a “CSF response signature” was seen in a subset of breast cancer that was associated with higher grade and decreased estrogen receptor expression [50]. Therefore, macrophages could play an important role in STS biology.

### Macrophages in sarcomas

Many characteristics of tumors are derived in varying degrees from infiltrating normal host cells, either stromal or blood-derived. Chronic inflammation has been linked to cancer development since the time of Virchow [48, 51], and tumors have been described as “wounds that do not heal” [52]; normal infiltrating inflammatory cells, including TAMs and even myofibroblasts, could contribute to tumor growth (reviewed in [18, 53–55]). In fact, infiltration of tumors by macrophages, in some cases representing 50% of the cells in the tumor, was recognized by Virchow in the nineteenth century, terming them lymphoreticular cells [2, 51]. In some tumors, the number and/or distribution of TAMs correlated with prognosis [56]. While lymphocytes were sometimes seen in the samples, there was no clear correlation with macrophage density.

Macrophage infiltration of STS has been previously reported [57–62], and CD68 expression has been reported to have prognostic significance in leiomyosarcoma [58, 59]; in addition, some leiomyosarcoma cells were shown to produce M-CSF in vitro. Prominent infiltration of many STS tumors was also recently reported in a phase II trial of PD-1 inhibition [62]. Thus, the prominent macrophage infiltrate of many sarcomas suggests a potential role for targeting tumor macrophages in some sarcomas, as has been suggested in some other tumors [18, 58–61, 63]. In our study, both pre- treatment and post-treatment CD68 staining in viable tumor correlated positively with the baseline SUVmax and negatively with percent viable tumor cells at resection (after chemotherapy). Post-treatment CD68 staining also correlated weakly positively with the post-treatment SUVmax. While the negative correlation of post-treatment CD68 staining with percent viable tumor cells in the resection specimen could reflect a treatment effect, the negative correlation of the pre-treatment CD68 staining with percent viable tumor cells after chemotherapy further suggests that infiltrating macrophages may play an important role in STS biology.

The role of paracrine signaling in tumors is dramatically evident in giant cell tumor of bone where RANKL secretion by tumor cells recruits normal osteoclasts to the tumor from circulating precursors [64]. Elimination of osteoclasts by a monoclonal antibody to RANKL results in dramatic effects on the neoplastic tumor cells as well, with associated clinical benefit, presumably by eliminating factors produced by the osteoclasts that are beneficial for growth and maintenance of the tumor phenotype of the neoplastic cells [64–66]. Analogous to these observations, interfering with signaling from CSF1 produced by the tumor cells in pigmented villonodular synovitis can markedly decrease macrophage infiltration and have striking beneficial effects [67–69]. TAM infiltration in some other sarcomas could possibly affect tumor cell biology in a similar manner, and targeting macrophages has shown anti-tumor activity in a model of leiomyosarcoma [70–72]. Thus, it is possible that infiltrating CD68 positive macrophages in some STS could produce factors that have a trophic effect on the tumor cells, and that chemotherapy effects on these macrophages could result in less production of tumor supporting factors. Another possibility could reflect primary damage to the tumor resulting in new antigens then recognized by the macrophages resulting in augmented immune destruction.

Similarly, TAMs and other stromal cells may be involved in a variety of processes critical to tumor development including immuno-suppression, angiogenesis, invasion, metastasis, response to chemotherapy and radiation therapy (reviewed in [73, 74]), and macrophage content has been correlated with prognosis in several cancers [73]. For example, TAMs could contribute to tumor growth by several mechanisms, including releasing factors that promote tumor cell growth and inhibiting the immune response to the tumor (reviewed in [54]).

Macrophages can secrete a variety of cytokines capable of paracrine signaling and potentially affecting tumor biology [75]; one study suggested that CSF-1 production with macrophage infiltration may promote neovascularization [72], and angiogenesis plays an important role in tumor growth [17]. In our study, CD31 staining was similar before and after chemotherapy in most cases evaluated. Pre-treatment CD31 staining was negatively correlated with post-chemotherapy SUVmax and weakly with percent viable tumor cells in the resection specimen, and it was positively correlated with change in tumor size (i.e., more tumor growth and higher baseline CD31).

In addition to the possible effects of inflammatory cells in the tumor microenvironment on tumor growth and angiogenesis, tumor macrophages may influence response to chemotherapy [56, 61, 76–78]. Depending on the model, depletion of TAMs increased or decreased the efficacy of chemotherapy [73]. In fact, there is evidence that some of the anti-tumor effects of trabectedin may be related to a reduction in inflammatory mediator production by tumor cells and by reducing infiltrating inflammatory cells such as macrophages [76–78].

The presence of large numbers of macrophages in some tumors also raises the question of their role in the findings of our study correlating changes in PET scan measurements after chemotherapy with outcome [15]. The positive correlation of both pre-and post-treatment CD68 staining with the pre- and post-treatment SUVmax, respectively, suggests that intra-tumor macrophages may contribute significantly to the observed SUVmax on PET imaging in some cases. An analogous case occurs in giant cell tumor of bone, where normal osteoclasts are recruited by RANKL secreted by tumor cells [64, 79]; osteoclasts contain large amounts of H+ transporting ATPase and may avidly take up FDG [80, 81]. PET activity falls rapidly following treatment with denosumab, an antibody to RANKL, which may reflect the change in energy use by osteoclasts rather than reflecting a change in activity of neoplastic cells [79]. Similarly, FDG uptake by tumor macrophages may play a role in changes in PET activity in some sarcomas and other solid tumors, and this may contribute to less correlation between PET findings and pathologic findings of tumor viability. This possibility is supported by the positive correlation between CD68 staining and SUVmax in both the pre-and post-chemotherapy specimens. Macrophage infiltration also may have a significant effect on tumor volume and thereby contribute to traditional measures of tumor size and response to therapy such as RECIST.

### Cancer stem cells in sarcomas

Our findings suggest that while CD133, ALDH1, and CD44 may be useful markers of CSC in epithelial tumors, their clinical utility in STS seems limited. CD133 was reported to be expressed in synovial sarcoma [36], where CD133 expression was found in 5/5 tumor samples and 3/3 cell lines with the percentage of cells expressing CD133 ranging from ~ 2 to ~ 20%. A recent study found that a CD133+ subpopulation of a synovial sarcoma cell line had several CSC properties, including self-renewal and resistance to chemotherapy [32]. CD133 expression has also been reported in other sarcomas [35, 37], and correlated with lung metastases and poor prognosis in osteosarcoma [82], and poor survival in embryonal rhabdomyosarcoma [11]. In the current study, no CD133 staining was observed in 5 synovial sarcomas. While only 2 STS cases in the current study stained for CD133, the changes in CD133 staining following chemotherapy were compatible with a decrease in “stemness” after treatment.

ALDH1 has been proposed to be a marker of both normal and cancer stem cells, and expression in breast cancer has been correlated with survival in some studies [6]. However, the utility of ALDH1 as a marker of CSCs is not clear in some solid tumors such as ovarian cancer [12–14]. Liu et al. found ALDH1 expression in ~ 1% of cells in the synovial sarcoma cell line SW982 [32]. In the current study, ALDH1 staining of tumor cells was detected in 11 of 30 pre-treatment biopsy samples and was similar before and after chemotherapy in half of the cases, while in some cases staining was higher and in some lower. Overall, patients whose pre-treatment ALDH1 staining intensity was high had worse survival outcomes, and patients whose post-treatment ALDH1 staining intensity was high had increased tumor growth; however, these correlations were weak. Thus, ALDH1 expression does not seem to be a useful marker of CSC in STS.

CD44 has been described as a marker of CSC in some carcinomas [6, 8]. In our study, CD44 expression was detected in tumor cells in 26 of 30 pre-treatment samples and was similar before and after chemotherapy. As with ALDH1, CD44 does not appear to be a useful CSC marker in STS.

## Conclusions

We conclude that CD133, CD44, and ALDH1 are not likely to be clinically useful markers of CSC in STS. In addition, ALDH1 and CD44 were strongly expressed by macrophages, which commonly infiltrate tumors. In some cases, prominent macrophage infiltration of the tumor was observed. These macrophages may play an important role in tumor biology and response to chemotherapy. Our results further support the suggestion of macrophage targeting agents in selected STS, possibly before the use of other chemotherapy or immunotherapy. In addition, infiltrating macrophages may contribute significantly to the observed results of our PET imaging study [15]. Synovial sarcomas exhibited lower CD68 and CD44 staining, and less macrophage infiltration than the other sarcomas in our study.

## Additional files

**Additional file 1: Table S1.** Patient and disease characteristics.

**Additional file 2: Table S2.** IHC staining of pre- and post-chemotherapy samples.

## Abbreviations

CSC: cancer stem cell
STS: soft tissue sarcoma
SUVmax: maximum standardized uptake value
PET-CT: fluoro-deoxyglucose positron emission tomography combined with computerized axial tomography
IHC: immunohistochemistry
UPS: undifferentiated pleomorphic sarcoma
TAM: tumor-associated macrophage
