# Supplementary material for: Effect of chemotherapy on cancer stem cells and tumor-associated macrophages in a prospective study of preoperative chemotherapy in soft tissue sarcoma
Source: J Transl Med. 2019 Apr 18;17:130. doi: 10.1186/s12967-019-1883-6 (PMC6471853; doi:10.1186/s12967-019-1883-6)
Supplement: Supplementary file 1 — Additional file 1: Table S1. Patient and disease characteristics. [file 12967_2019_1883_MOESM1_ESM.docx]

| **Table S1: Patient and Disease Characteristics** | | | | |
| --- | --- | --- | --- | --- |
| **Case** | **Sex** | **AJCC Stage** | **Histology** | **% Viable** |
| 1 | F | 3 | Liposarcoma with pleomorphic components | 4 |
| 2 | F | 3 | UPS | 2 |
| 3 | M | 4 | Synovial sarcoma | 0 |
| 4 | M | 3 | Synovial sarcoma | 50 |
| 5 | M | 3 | UPS | 0 |
| 6 | F | 3 | UPS | 0 |
| 7 | M | 3 | Monophasic synovial sarcoma | 10 |
| 8 | M | 3 | UPS | 1 |
| 9 | M | 4 | High-grade sarcoma with epitheloid features | 0 |
| 10 | M | 3 | malignant peripheral nerve sheath tumor | 4 |
| 11 | F | 3 | Biphasic synovial sarcoma | 100 |
| 12 | M | 3 | Fibrosarcoma | 1 |
| 13 | F | 3 | Dedifferentiated Liposarcoma | 0 |
| 14 | F | 3 | Monophasic Synovial sarcoma | 30 |
| 15 | F | 3 | UPS | 80 |
| 16 | F | 4 | Leiomyosarcoma | 7 |
| 17 | M | 3 | UPS | 5 |
| 18 | F | 4 | UPS | 20 |
| 19 | F | 3 | UPS | 1 |
| 20 | F | 3 | Fibrosarcoma | 70 |
| 21 | M | 3 | UPS | 25 |
| 22 | M | 3 | UPS | 0 |
| 23 | M | 3 | UPS | 25 |
| 24 | F | 3 | UPS | 0.5 |
| 25 | F | 4 | UPS | 0 |
| 26 | F | 3 | Dedifferentiated Liposarcoma | 10 |
| 27 | F | 3 | UPS | 2 |
| 28 | M | 3 | UPS | 30 |
| 29 | F | 4 | UPS | 10 |
| 30 | M | 3 | UPS | 70 |
| 31 | M | 3 | Spindle cell sarcoma | 10 |
| **Abbreviations**: AJCC, American Joint Committee on Cancer; UPS, undifferentiated pleomorphic sarcoma.  % viable is % viable tumor cells in the post-chemotherapy resection specimen | | | | |
