# Supplementary material for: Effect of chemotherapy on cancer stem cells and tumor-associated macrophages in a prospective study of preoperative chemotherapy in soft tissue sarcoma
Source: J Transl Med. 2019 Apr 18;17:130. doi: 10.1186/s12967-019-1883-6 (PMC6471853; doi:10.1186/s12967-019-1883-6)
Supplement: Supplementary file 2 — Additional file 2: Table S2. IHC staining of pre- and post-chemotherapy samples. [file 12967_2019_1883_MOESM2_ESM.docx]

| **Table S2: IHC staining of pre- and post-chemotherapy samples** | | | | | | | | | |
| --- | --- | --- | --- | --- | --- | --- | --- | --- | --- |
| **Case** | **Sample** | **CD68** | **ALDH1** | | **CD44** | | **CD133** | **CD31** | **SUV*** |
|  |  | **%pos** | **%pos** | **Int** | **%pos** | **Int** | **%pos** |  |  |
| 1 | Pre | QNS | 0 | 0 | 3 | 2 | QNS | QNS | 18.1 |
|  | Post | 25-50 | QNS | QNS | 2 | 2 | 0 | 3 | 3.31 |
| 2 | Pre | 25-50 | 0 | 0 | 3 | 3 | 0 | 1 | 18.8 |
|  | Post | 50-75 | 1 | 2 | 3 | 3 | 0 | 1 | 8.5 |
| 3 | Pre | 25-50 | 0 | 0 | 1 | 2 | 0 | 2 | 15.3 |
|  | Post | QNS | QNS | QNS | QNS | QNS | QNS | QNS | 0.8 |
| 4 | Pre | 5-25 | 0 | 0 | 0 | 0 | 0 | 2 | 9.4 |
|  | Post | 0-5 | 1 | 2 | 2 | 2 | 0 | 2 | 2.7 |
| 5 | Pre | 25-50 | 0 | 0 | 2 | 2 | 0 | 3 | 7.7 |
|  | Post | 25-50 | QNS | QNS | 3 | 2 | 0 | 3 | 0.8 |
| 6 | Pre | 50-75 | 1 | 1 | 3 | 2 | 0 | 2 | 41.6 |
|  | Post | QNS | QNS | QNS | QNS | QNS | QNS | QNS | 4.4 |
| 7 | Pre | 0-5 | 0 | 0 | 1 | 1 | QNS | 3 | 6.7 |
|  | Post | 5-25 | 0 | 0 | 1 | 1 | 0 | 3 | 0.8 |
| 8 | Pre | 5-25 | 0 | 0 | 1 | 1 | 0 | 2 | 20.8 |
|  | Post | 75-100 | QNS | QNS | QNS | QNS | 0 | 3 | 10.8 |
| 9 | Pre | QNS | QNS | QNS | QNS | QNS | QNS | QNS | 22 |
|  | Post | QNS | QNS | QNS | QNS | QNS | QNS | QNS | 4 |
| 10 | Pre | 0-5 | 2 | 1 | 3 | 2 | 1 and 3 ** | 1 | 13.9 |
|  | Post | 25-50 | QNS | QNS | 1 | 1 | 1 ^✝^ | 2 | 5.6 |
| 11 | Pre | 0-5 | 3 | 2 | 2 | 2 | 0 | 1 | 5.5 |
|  | Post | QNS | QNS | QNS | QNS | QNS | QNS | QNS | 4.8 |
| 12 | Pre | 25-50 | 1 | 1 | 2 | 1 | 0 | 3 | 4.9 |
|  | Post | 50-75 | 1 | 1 | 2 | 1 | 0 | QNS | 2.5 |
| 13 | Pre | 25-50 | 0 | 0 | 3 | 3 | 0 | 2 | 12.6 |
|  | Post | QNS | QNS | QNS | QNS | QNS | QNS | QNS | 4 |
| 14 | Pre | 5-25 | 0 | 0 | 0 | 0 | 0 | 1 | 1.2 |
|  | Post | 0-5 | 0 | 0 | 1 | 2 | 0 | 2 | 0.8 |
| 15 | Pre | 5-25 | 1 | 1 | 3 | 2 | 0 | 2 | 11 |
|  | Post | QNS | QNS | QNS | QNS | QNS | QNS | QNS | 5.4 |
| 16 | Pre | 0-5 | 0 | 0 | 0 | 0 | 0 | 2 | 5.3 |
|  | Post | 25-50 | 1 | 2 | 0 | 0 | 0 | 2 | 4.2 |
| 17 | Pre | 50-75 | 0 | 0 | 3 | 2 | 0 | 2 | 21.8 |
|  | Post | 50-75 | QNS | QNS | 3 | 2 | 0 | 2 | 5.6 |
| 18 | Pre | 25-50 | 0 | 0 | 3 | 3 | 0 | 2 | 27.4 |
|  | Post | 50-75 | QNS | QNS | 3 | 3 | 0 | 2 | 25.6 |
| 19 | Pre | 5-25 | 1 | 2 | 2 | 1 | 1 ^✝✝^ | 2 | 4 |
|  | Post | 50-75 | QNS | QNS | 2 | 1 | 0 | 2 | 2.3 |
| 20 | Pre | 5-25 | 1 | 1 | 2 | 1 | 0 | 2 | 11 |
|  | Post | 25-50 | 1 | 3 | 2 | 2 | 0 | 2 | 4.5 |
| 21 | Pre | 5-25 | 0 | 0 | 1 | 1 | 0 | 2 | 14.9 |
|  | Post | 25-50 | 0 | 0 | 3 | 3 | 0 | 2 | 3.2 |
| 22 | Pre | 25-50 | 0 | 0 | 0 |  | 0 | 1 | 9.4 |
|  | Post | 75-100 | 0 | 0 | 3 | 2 | 0 | 2 | 3.9 |
| 23 | Pre | 50-75 | 3 | 1 | 3 | 2 | 0 | 2 | 11.9 |
|  | Post | 50-75 | 2 | 1 | 3 | 2 | 0 | 2 | NA |
| 24 | Pre | 50-75 | 0 | 0 | 3 | 3 | 0 | 2 | 18.5 |
|  | Post | 50-75 | 0 | 0 | 3 | 3 | 0 | 1 | 3.0 |
| 25 | Pre | QNS | 2 | 2 | 2 | 2 | QNS | QNS | 15.1 |
|  | Post | QNS | QNS | QNS | 3 | 2 | QNS | QNS | 5 |
| 26 | Pre | 25-50 | 0 | 0 | 3 | 2 | 0 | 2 | 4 |
|  | Post | QNS | QNS | QNS | 3 | 2 | QNS | QNS | 5.7 |
| 27 | Pre | 5-25 | 3 | 1 | 3 | 3 | QNS | 2 | 4.8 |
|  | Post | 50-75 | 0 | 0 | 3 | 3 | 0 | 2 | 2.1 |
| 28 | Pre | 25-50 | 0 | 0 | 3 | 3 | 0 | 1 | 5.3 |
|  | Post | 25-50 | QNS | QNS | 3 | 2 | 0 | 1 | 9.1 |
| 29 | Pre | 25-50 | 0 | 0 | 3 | 3 | 0 | 1 | 22.6 |
|  | Post | 50-75 | QNS | QNS | 3 | 3 | 0 | 1 | NA |
| 30 | Pre | 25-50 | 0 | 0 | 3 | 2 | 0 | 1 | 3.2 |
|  | Post | 50-75 | QNS | QNS | 3 | 2 | 0 | 2 | 4.4 |
| 31 | Pre | 5-25 | 2 | 1 | 2 | 2 | QNS | QNS | 9.1 |
|  | Post | 25-50 | 0 | 0 | 3 | 2 | 0 | 2 | 5.2 |
| Abbreviations: %pos, percent positive; Int, staining intensity; Pre, pre-chemotherapy; Post, post-chemotherapy; QNS, quantity not sufficient  * SUV was performed before chemotherapy (Pre) and after 4 cycles of chemotherapy (Post).  ** 66-100% tumor cells low positive (1) and 33% high positive (3) tumor cells  ✝ 1-33% low positive (1/1) among cells with retained spindle characteristics; more epithelioid and mulinucleated negative  ✝✝ 33% tumor low positive at 1 (1/1) | | | | | | | | | |
